# Predictive value of indicator of CA125 combined with D-dimer (ICD) for lymph node metastasis in patients with ovarian cancer: A two center cohort study

**DOI:** 10.7150/jca.70737

**Published:** 2022-05-01

**Authors:** Li Zhang, Zongyu Guan, Yi Yin, Chaoyang Ou, Hongyan Qian, Min Tang, Aiguo Shen

**Affiliations:** 1 Department of Cancer Research Center, Nantong Tumor Hospital, The Affiliated Tumor Hospital of Nantong University, Nantong 226361, China.; 2 Department of Obstetrics and Gynaecology, Nantong University, Nantong 226361, China.; 3 Department of Clinical Laboratory Diagnostics, Nantong University, Nantong 226361, China.; 4 Department of Oncology, Nantong University, Nantong 226361, China.

**Keywords:** ovarian cancer (OC), CA125, D-dimer, lymph node metastasis (LNM)

## Abstract

**Background:** The clinical serum markers CA125 and D-dimer have been reported to predict lymph node metastasis(LNM) in several malignant tumors, but the reports in ovarian cancer(OC) are still absent. The purpose of this study was to explore the value of indicator CA125 combined with D-dimer (ICD) in predicting LNM in patients with OC.

**Methods:** A total of 447 patients diagnosed with OC from January 2008 to June 2019 were included in this retrospective study as the training set. A total of 284 patients were included in the validation set. The optimal cut-off critical value of ICD was evaluated by the receiver operating characteristic curve (ROC), and the maximum Youden index (sensitivity + specificity-1). Univariate and multivariate analysis were used to evaluate ICD as a predictor of LNM in OC.

**Results:** According to ROC curve, area under curve (AUC) of ICD (AUC=0.706, p<0.001) was significantly larger than that of CA125 (AUC=0.671, p<0.001) and D-dimer (AUC=0.562, p=0.022) alone. Multivariate analysis showed that ICD (HR 2.651, 95% CI 1.273-5.520, p=0.009) was an independent predictor of LNM and overall survival (OS) in OC. It has also been verified in another medical center.

**Conclusion:** ICD is an independent predictor of LNM in ovarian cancers, which is helpful for clinicians to draw up individual treatment plans.

## Introduction

The incidence rate of ovarian cancer (OC) is the third place in gynecologic malignancies, which is only the next to cervical and endometrial cancer. However, the mortality rate has always been the highest in female reproductive system malignancies 1. Because of its special anatomical location in the pelvic cavity, the pathogenesis is not fully understood, and there are no obvious symptoms in the early stage 2. Therefore, although the 5-year survival rate of early stage (FIGO stage I and II) OC can reach 90% through surgery and chemotherapy, the early diagnosis rate is only 25%. More than 70% of OC patients are found to be advanced (FIGO stage III and IV), resulting in poor outcomes and the 5-year survival rate is only 29% 3-4. If the staging status of OC can be accurately predicted in advance, it is of great significance for the treatment of advanced OC. It can not only reduce the mortality of advanced OC, but also improve the OS rate.

Lymph node metastasis (LNM) as the major mode of malignant tumor diffusion, is the main way of OC metastasis, and also an important reason for poor prognosis 5. The data showed that the 5-year survival rate of OC patients with LNM was lower than 40% of patients without metastasis 6. It is reported that neoadjuvant therapy will be given priority to OC patients with LNM in order to reduce the tumor size and/or eliminate the invisible metastatic cells, so as to improve the survival rate and prolong the recurrence time of patients 7. As a result, accurate preoperative prediction of lymph node status is of great significance for the treatment of OC.

Endoscopic ultrasonography (EUS), computed tomography (CT), magnetic resonance imaging (MRI), positron emission tomography (PET) and other imaging techniques are widely used to evaluate the status of lymph nodes in patients with OC 8. However, their application is limited due to the inconsistency of sensitivity and specificity, high radiation, high examination cost and noise. Some new serum biomarkers, such as AEG-1, MAC30 and Rspo3, have been proposed to detect LNM in ovarian cancers 9-11. However, it is difficult to achieve clinical application due to the high cost and complex technology.

Cancer antigen 125(CA125) also named MUC16A is a mature serum tumor marker, which plays a crucial role in cell adhesion, invasion and metastasis, and enhances the malignant potential of OC 12. Recognized in clinical, serum CA125 level is primarily used for early diagnosis, preoperative evaluation, chemotherapy response evaluation and follow-up of patients with pelvic masses 13-14. Generally, the normal level of serum CA125 should be less than or equal to 35U/ ml, while considered to be related to malignant tumor disease if it is higher than this value. Therefore, this critical value is often used in clinical trials. In view of the poor sensitivity and accuracy of serum CA125 as a single indicator to evaluate LNM of OC 15, we aimed to seek a new serum indicator combined with it to improve the accuracy of predicting LNM of OC.

D-dimer is the fibrin which is degraded from the fibrinolytic system activated by fibrinolytic protein. In recent years, it has been investigated that D-dimer can participate in the progress and spread of malignant tumors through a variety of mechanisms, including colorectal liver metastasis, mediastinal lymph node invasion of non-small cell lung cancer and cervical cancer LNM 16-19. These findings indicated that elevated D-dimer level can be applied as an effective indicator to predict the metastasis of malignant tumors. To our knowledge, there is no report about the potential role of D-dimer level in predicting LNM of OC. Since LNM is one of the most important event in clinical decision-making of OC, we established a new parameter based on preoperative D-dimer and CA125, and evaluated the predictive performance of these two serum markers. This has better predictive value for LNM in OC patients, which may help in drawing up surgical strategies and follow-up plans.

## Materials and Methods

### Study population

We retrospectively analyzed the clinical data of OC patients who were diagnosed in Affiliated Tumor Hospital of Nantong University and Affiliated Hospital of Nantong University from January 2008 to June 2019. The diagnostic criteria were determined according to the latest NCCN guidelines for OC diagnosis and treatment. We only included OC patients who underwent primary tumor reduction surgery for histopathological examination and identified by three pathologists to ensure the results. CA125, D-dimer and HE4 were measured one week before laparotomy. Clinicopathological data collected included age, menopausal status, International Federation of Gynecology and Obstetrics (FIGO) stage, histological subtype, degree of tumor cell differentiation, ascites, LNM and OS. OS was defined as the time from diagnosis to death of OC, or the time to the last follow-up if the patient was still alive. The ultimate follow-up date was June 1, 2021. According to some irresistible factors, the exclusion criteria are as follows: 1. with incomplete clinical serological data (CA125, D-dimer and HE4); 2. with incomplete pathological data; 3. with previous cancer history or multiple cancers at present; 4. with diseases that could affect CA125 or D-dimer. This study was approved by the Ethics Committee of Tumor Hospital Affiliated of Nantong University and Affiliated Hospital of Nantong University, and carried out in accordance with relevant guidelines and regulations. The study was conducted in conjunction with the Helsinki Declaration (revised in 2013). Finally, 447 and 284 eligible patients were enrolled in the two medical centers.

### Data calculation

Principle of calculating the ICD value is based on the logistic regression. According to the logistic regression principle, the logistic function is defined as:



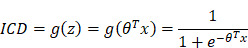



to represent the prediction of LNM, value of which must be within 0~1. And the decision boundary is defined as:



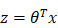





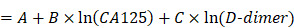





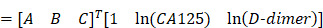



Furthermore, for the logistic regression, the threshold of ICD is set as 50%, which corresponds to *z* =0. Using these functions, if *z* is more than 0, then ICD will be more than 50%, which causes that LNM is predicted. On the contrary, if *z* is less than 0, then ICD will be less than 50%, which causes that non-LNM is predicted.

Hence, when using the logistic regression, the coefficient matrix [A, B, C] of the decision boundary is very important. Building the cost function and using the gradient descent can find the coefficient matrix [A, B, C]. To obtain the coefficient matrix *θ* = [A, B, C], the training set of *m* samples is used, written as {(*x_1_, y_1_*), (*x_2_, y_2_*) … (*x_m_, y_m_*)}, where *x* and *y* represent indictors and the actual LNM result of training samples, respectively. The cost function is built:



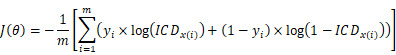



Using the gradient descent to iterate so as to solve out the *θ* which makes the cost minimum, written by:



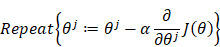





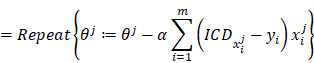



where *α* is the learning step.

As a result, the coefficients *θ* = [A, B, C] of each age group are obtained according the above formula. Furthermore, the ICD formula will be used to predicate patients' LNM.

In this paper, the used coefficients *θ* = [A, B, C] of each age group are given as follows:

- Years below 31: A= (-6.5761); B=1.6183; C=1.7050,
- Years from 31 to 40: A= (-4.6442); B=0.6059; C=0.6626,
- Years from 41 to 50: A= (-3.4778); B=0.6034; C=0.1127,
- Years from 51 to 60: A= (-2.1286); B=0.3483; C= (-0.2763),
- Years from 61 to 70: A= (-0.6698); B=0.1397; C=0.3494,
- Years from 71 to 80: A= (-1.7603); B=0.3757; C= (-0.4508),
- Years above 80: A= 0.6698; B=1.5957; C= (-9.0530).

### Statistical analysis

All the statistical analysis was carried out by International Business Machines Corporation Statistical Package for the Social Sciences (IBM SPSS Statistics) software (Version 25.0). The predictive performance of CA125, D-dimer and ICD was evaluated by ROC, and the maximum Youden index (sensitivity + specificity-1) was selected as the optimal cut-off critical value. The chi-square test and Fisher' s exact probability test were used to compare categorical variables, and the Kaplan- Meier method organized for evaluating differences in OS. Univariate and multivariate logistic regression models were employed to identify the independent predictors of LNM. Results were exhibited as hazard ratio (HR) and 95% confidence interval (CI). Finally, Forest Plot had been drawn using the R programming language. The value of p<0.05 was considered statistically important.

## Results

### Patient characteristics

**Figure 1:**
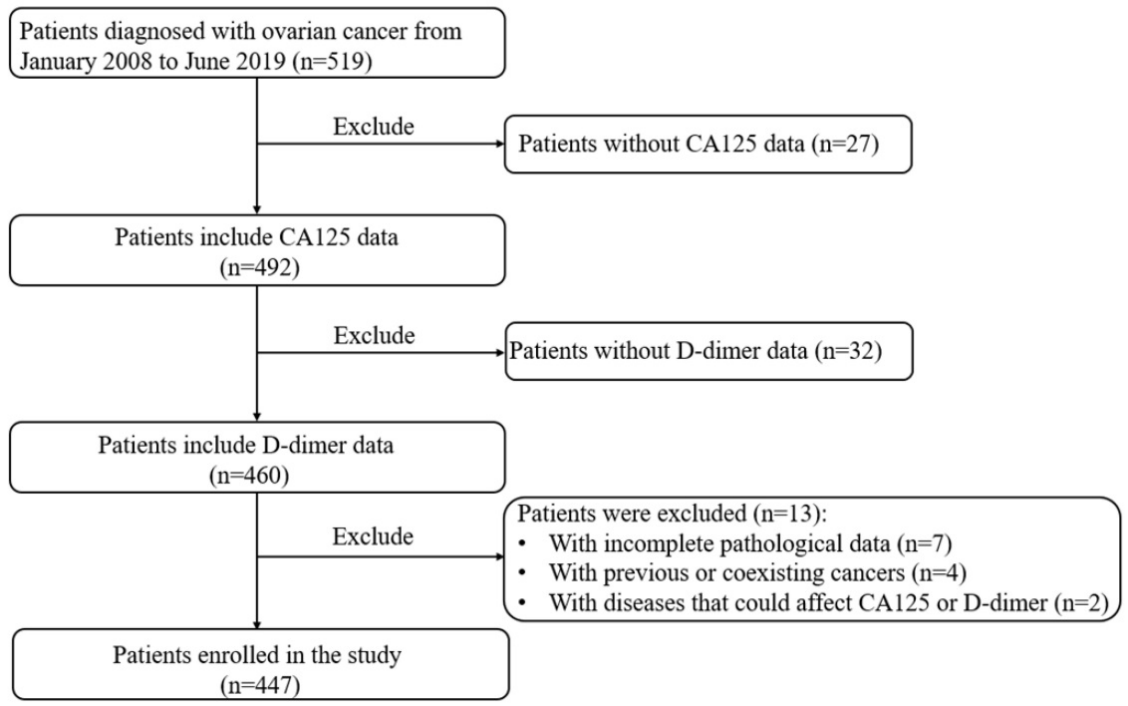
** Flow chart of patient selection.** Abbreviations: CA125, cancer antigen 125.

A total of 447 ovarian cancers from Affiliated Tumor Hospital of Nantong University were qualified for the study (Fig. 1). Detailed and clinical factors of patients are summarized in Table 1. Median age at diagnosis of a patient was 58 years (age from 16 to 87). All patients were divided into 7 groups according to disparate ages. See Table 1 for details. Taking the physiological status of the patients into consideration, 115 (25.73%) of them were premenopausal, 307 (68.68%) were postmenopausal, and 25 (5.59%) were hysterectomy patients. On the basis of FIGO stage, 104 (23.27%) patients were with stage I-II tumors, 343 (76.73%) were with stage III-IV tumors. 151 (33.78%) patients were detected to have serous histology whereas 296 (66.22%) had non-serous histology. LNM was detected in 231 (51.68%) patients. The distribution of ascites, D-dimer, CA125 and degree of differentiation were presented in Table 1.

### Univariate and Multivariate analysis for LNM related factors of ovarian cancers

**Figure 2:**
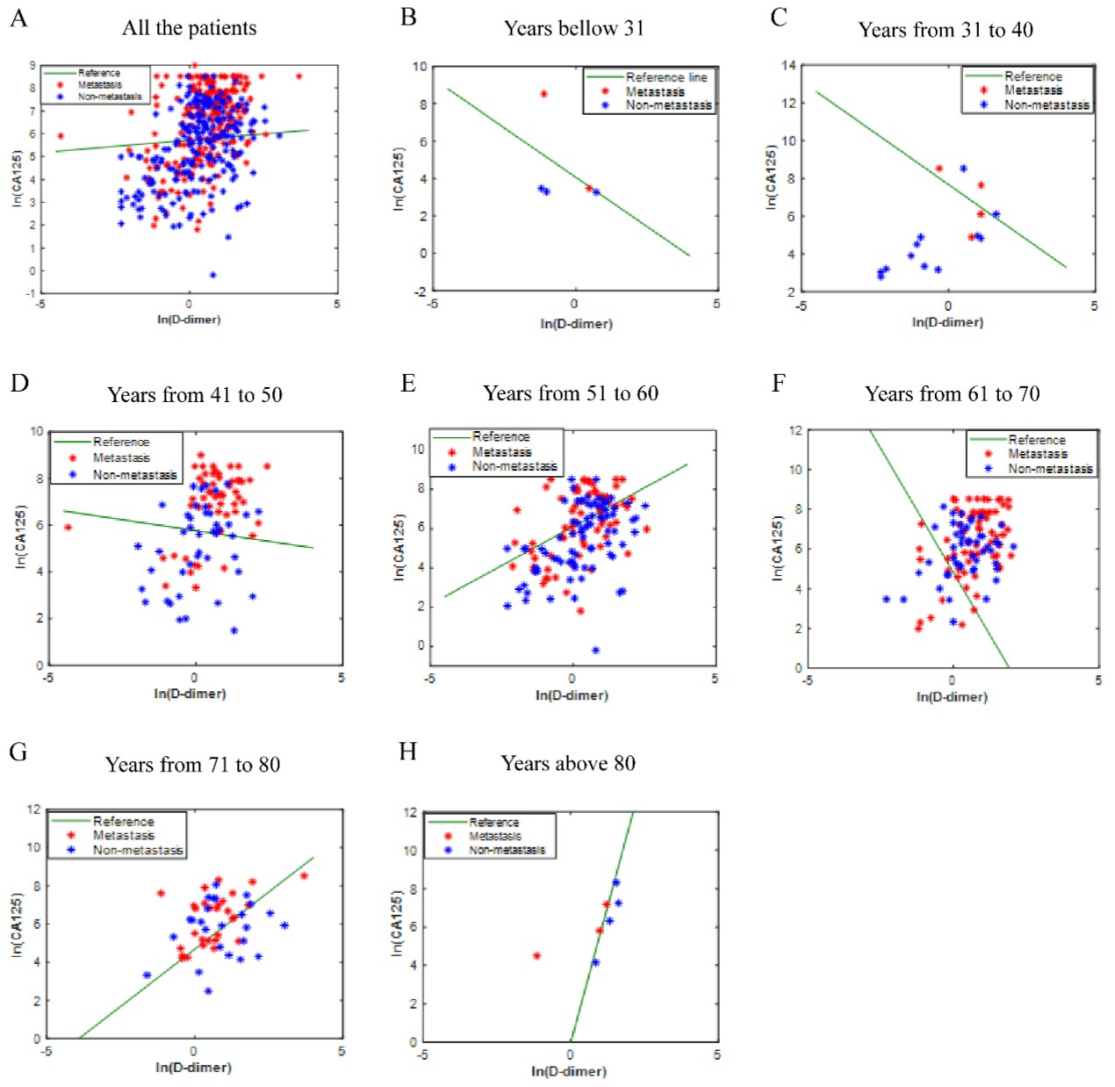
** The ICD efficiency chart of all (A) and classified (B-H) patients.** The points on the graph represent the calculated ICD values of each individual. There is no transfer below the reference line; meanwhile, there is transfer above the reference line. Abbreviations: CA125, cancer antigen 125; ICD, indicator of CA125 combined with D-dimer.

The values of CA125 and D-Dimer of all patients were fitted to obtain the ICD value based on the principle of logistic regression, but there is no rule to follow (Fig. 2A). Considering that ovarian disease is closely related to women's age, all patients were divided into seven age groups. The fitted values were displayed in Fig. 2B-2H. As shown in the figures, the fitting effect is relatively respectable except for years from 61 to 70.

Univariate analysis revealed that serum HE4 ≥ 320 pmol/L (HR: 4.056, 95% CI: 1.944-8.465, p<0.001), serum CA125 ≥ 490 U/mL (HR: 2.329, 95% CI: 1.593-3.403, p<0.001), serum D-dimer ≥ 1.69 mg/L (HR: 1.611, 95% CI: 1.109-2.341, p =0.012), ICD index > 0.5 (HR: 2.841, 95% CI: 1.917-4.129, p<0.001) and FIGO stage (HR: 2.901, 95% CI: 1.288-6.534, p=0.009) were independent risk factors for LNM in ovarian cancers (Table 2).

In the multivariate analysis, greater CA125 (HR: 0.408, 95% CI: 0.170-0.977, p=0.044), together with D-dimer (HR: 1.897, 95% CI: 1.025-3.513, p=0.042), ICD (HR: 2.651, 95% CI: 1.273-5.520, p=0.009), and FIGO stage (HR: 2.891, 95% CI: 1.291-6.473, P=0.01) were significant predictors for LNM (Table 2). The HE4 was null and void predictor of LNM (P>0.05; Table 2).

### Comparison of ROC curve of CA125, D-dimer, as well as ICD in predicting LNM rather than OS

**Figure 3:**
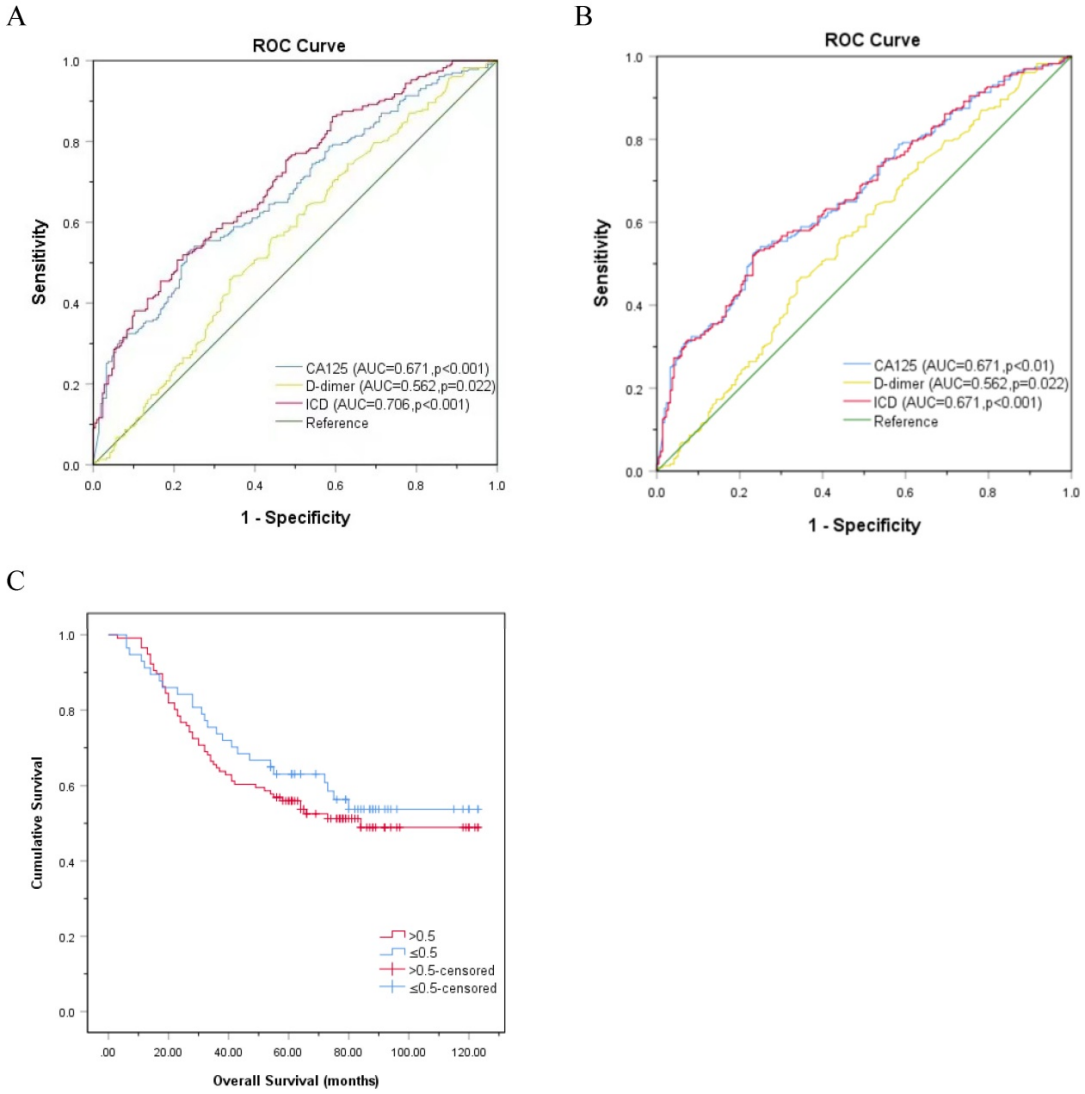
** ROC curve analysis based on CA125, D-dimer, and ICD for LNM. A.** Classified by age, p<0.001. **B.** Not classified by age, p<0.001. **C.** Kaplan-Meier curve for OS in patients with OC according to ICD, the reference threshold is 0.5, p>0.05. Abbreviations: CA125, cancer antigen 125; ICD, indicator of CA125 combined with D-dimer.

ROC curve was used to evaluate CA125, HE4, and ICD in the entire study population. Figure 3A exhibits that the AUC of CA125 (AUC=0.671, p<0.001) and D-dimer (AUC=0.562, p=0.022) were narrower than ICD (AUC=0.706, p<0.001), and the sensitivities (specificities) were 52.4% (76.4%), 45.9% (66.2%), 50.6% (79.2%), respectively, which indicated that the ability of ICD value to distinguish LNM is more meaningful than individual indicators of CA125 and D-dimer (Fig. 3A). When comparing the ICD (AUC = 0.671, p<0.001) before age grouping with CA125 (AUC = 0.671, p<0.001) and D-dimer (AUC = 0.562, p=0.022), superiority of ICD was not observed in predicting LNM (Fig. 3B). The association between ICD and OS was also showed by Kaplan-Meier curve (Fig. 3C). The result certified that even if the association between ICD and OS is meaningless, there is a trend that high expression of ICD is positively correlated with poor prognosis (p>0.05).

### Analysis of ICD in independent validation set from another center

**Figure 4:**
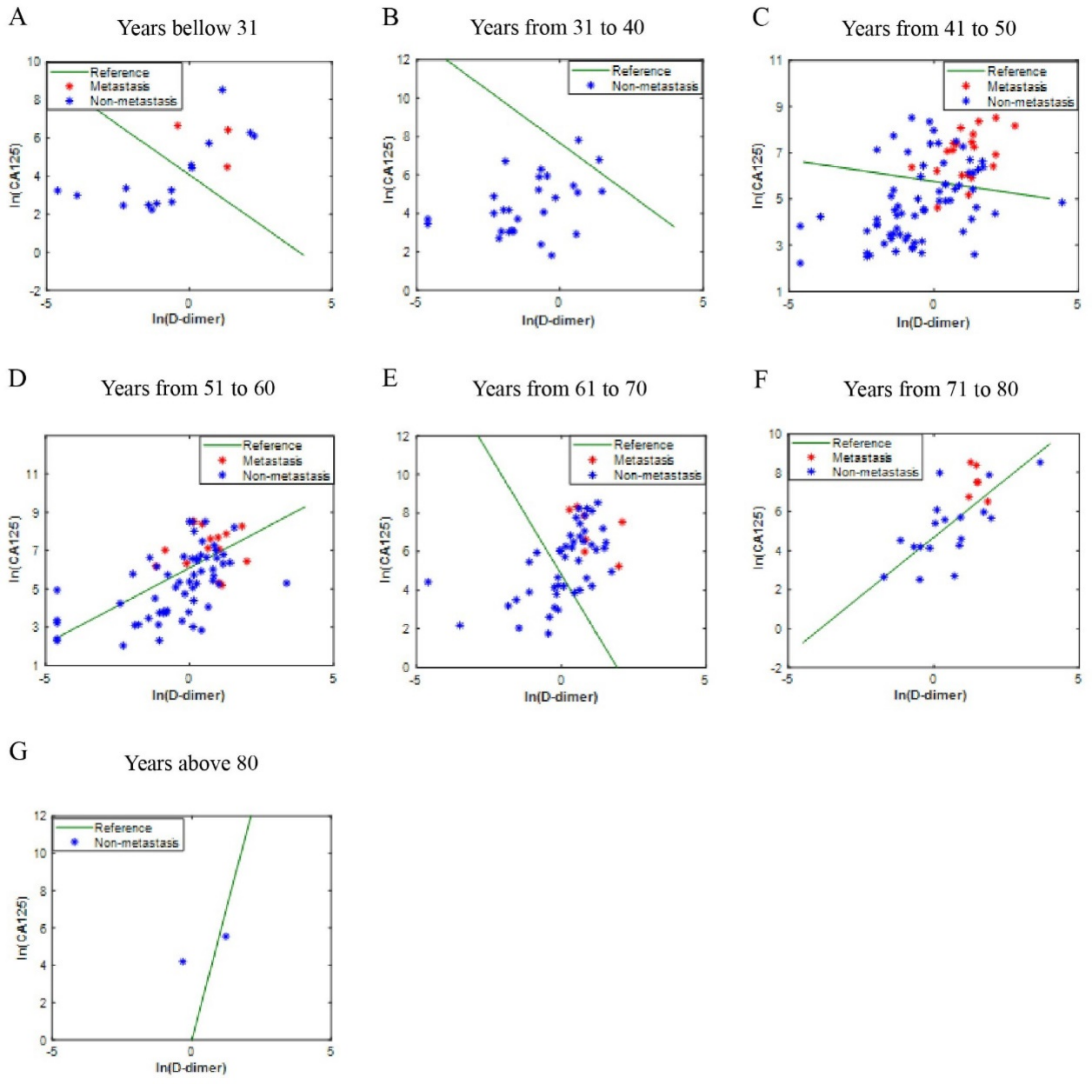
** Another center verification of ICD efficiency.** The points on the graph represent the calculated ICD values of each individual. There is no transfer below the reference line; meanwhile, there is transfer above the reference line; **A-G** are different age groups. Abbreviations: CA125, cancer antigen 125; ICD, indicator of CA125 combined with D-dimer.

**Figure 5:**
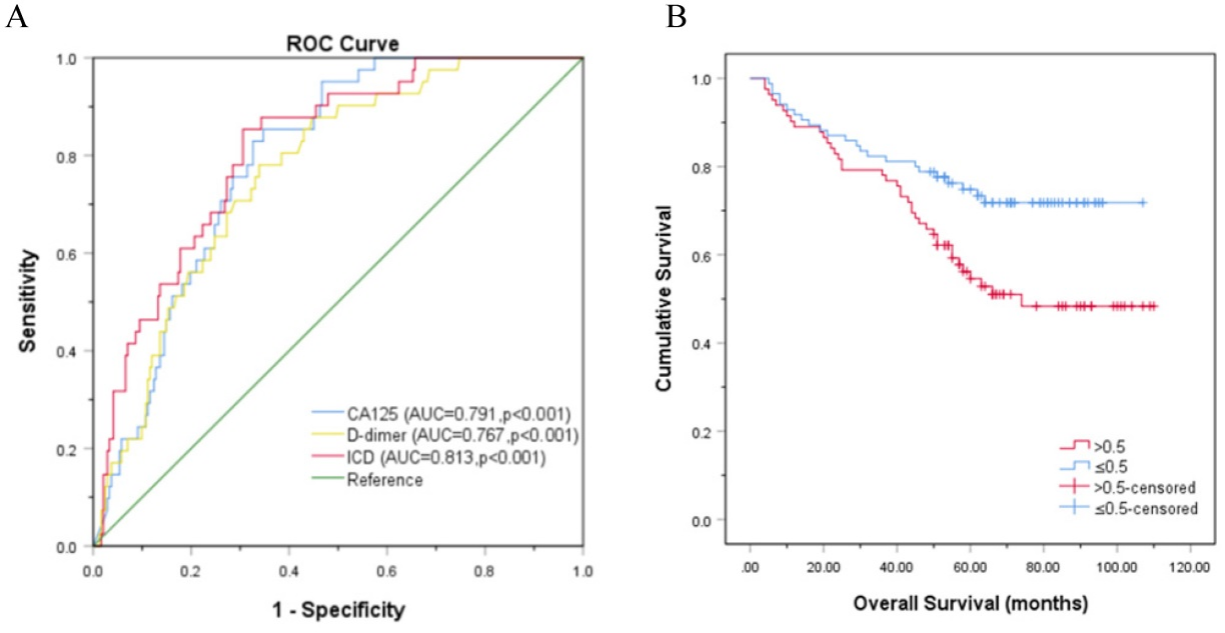
** Another center verification of ROC and Kaplan-Meier curve. A.** Another center verification of ROC curve analysis based on CA125, D-dimer, and ICD for LNM, p<0.001; **B.** Kaplan-Meier curve for OS in patients with OC according to ICD, the reference threshold is 0.5, p<0.05. Abbreviations: CA125, cancer antigen 125; ICD, indicator of CA125 combined with D-dimer.

The independent validation set was composed of patients who were diagnosed with OC in the Affiliated Hospital of Nantong University. In this validation set, most of the blue dot patients without LNM were below the reference line, while most of the red dot patients with LNM were above the reference line (Fig. 4A-4G). Moreover, the ICD (AUC = 0.813, p<0.001) is superior to CA125 (AUC = 0.791, p<0.001) and D-dimer (AUC = 0.767, p<0.001) in predicting LNM, the sensitivities (specificities) were 85.4% (65.3%), 78.0% (66.1%), 85.4% (69.4%), respectively (Fig. 5A). Kaplan-Meier curve showed that ICD was positively correlated with poor prognosis, and the difference was statistically significant (Fig. 5B) (p<0.05).

### Forest Plot shows the results of multivariate regression analysis

**Figure 6:**
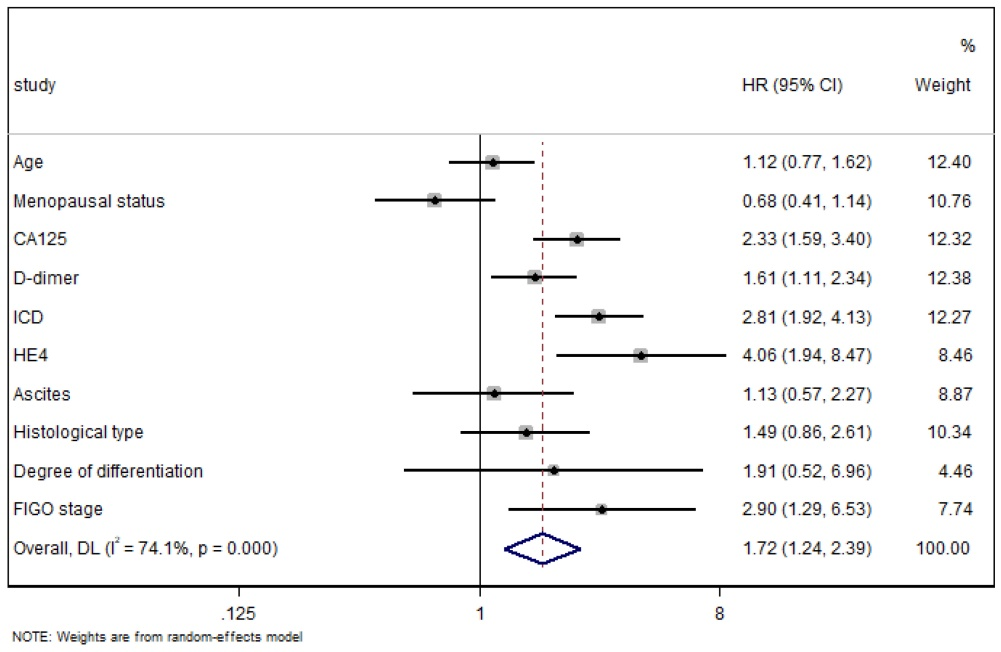
** The weight of the factors affecting lymph node metastasis.** Abbreviations: HE4, human epididymis secretory protein 4; CA125, cancer antigen 125; FIGO, International Federation of Gynecology and Obstetrics; ICD, indicator of CA125 combined with D-dimer.

All the indexes were summarized in the Forest Plot of association to LNM in OC. A random-effect model was used for distinct heterogeneity among indexes (I^2^=74.1%, P<0.01) (Fig. 6), and patients with high ICD tended to undergo LNM compared with that with low ICD (HR=2.81, 95%CI=1.92-4.13, P<0.01).

## Discussion

Our study exhibit that 51.68% (231/447) ovarian cancers had LNM when diagnosed. As an independent prognostic factor for OS, LNM is demonstrated by many previous researches 20-21 and summed in a meta-analysis 22. As an indicator of poor prognosis, it is also a key factor for patients with OC to select neoadjuvant therapy before surgery 23-24. Therefore, it is of great importance to seek the preoperative predictors of LNM in OC patients for the follow-up treatment plan.

It has been reported that cancer cells can activate the coagulation system and induce low-grade disseminated intravascular coagulation (DIC) or venous thromboembolism (VTE) 25. Therefore, the hypercoagulable state of many cancers is strongly related to tumor progression 26-27. D-dimer is an indicator of the formation and degradation of intravascular fibrin. When plasmin induced fibrinolysis system is activated, it comes from degraded fibrin. The specific reasons for the high expression of D-dimer are: when patient malignant tumor cells induce the release of tissue factors involved in the assembly of coagulation complex and the shedding of circulating coagulation promoting microbubbles, the expression of coagulation initiation protein, and the surface exposure of phosphatidylserine, these lead to the increased expression of D-dimer 28-29. In 2018, Ghadhban, BR et al. proved that plasma D-dimer is positively correlated with the progression and prognosis of breast cancer 30. In addition, the elevated D-dimer levels were associated with poor prognosis in patients with epithelial ovarian, renal, and lung cancer in several retrospective studies 31-33. Recently, the preoperative D-dimer level can predict the major complications or survival of patients with colorectal cancer liver metastasis after hepatectomy, and it also indicates that the D-dimer level is related to the prognosis of colorectal cancer 34. However, there are few studies on the potential role of plasma D-dimer level in predicting lymph node involvement in cancer patients. In 2000, Blackwell et al. demonstrated that the elevated plasma D-dimer indicates the positive involvement of axillary lymph nodes in breast cancer 35. Subsequently, plasma D-dimer levels were positively correlated with lymph node involvement in esophageal and gastric cancer 36-37. In recent years, two retrospective studies have reported the significant relationship between plasma D-dimer level and malignant LNM in lung cancer and cervical cancer 18-19.

In our study, we combined OC specific tumor marker CA125 with plasma D-dimer, and establish a new parameter ICD according to the age of patients to improve the predictive value. CA125 is a classic diagnostic biomarker of OC 38, but its value in predicting LNM of malignant tumor has rarely been studied. To the best of our knowledge, this is the first study to assess the association between ICD and LNM in OC. In our retrospective study, ROC curve demonstrated that ICD is strongly linked to LNM, and its prediction capacity is stronger than any single CA125 and D-dimer parameters. Furthermore, it was validated at another medical center. It is worth noting that the results of our study showed that tumor differentiation was not a predictor of LNM in univariate analysis. This is in contrast to the results of previous studies on other malignant tumors, which may be related to the large difference in the distribution of the degree of differentiation in the samples. In our study, we also found that ICD was associated with OS. Next, we will use ICD to attempt to predict OS to help doctors identify patients with poor prognosis and provide individual treatment.

In clinic, the main methods to evaluate LNM in cancer include endoscopy, laparotomy and imaging. Although the accuracy of endoscopic and laparotomy is high 39, the patient compliance is low as these techniques are invasive. Although less invasive imaging techniques, such as B-ultrasound, CT, PET and MRI, can accurately detect and evaluate the status of lymph nodes, but these methods are costly and have high ionizing radiation 40. In contrast, blood tests are relatively noninvasive, convenient, economical, effective and repeatable, and free of ionizing radiation. Blood tests of D-dimer and CA125 may be ordinary indicators to decide whether further particular imaging examination is demanded. In addition, serum biomarkers are more appropriate to long-term follow-up. In the future, we will investigate the predictive value of imaging technology combined with this index for LNM. The ICD index in our study has very practical clinical significance. Prior to surgery, the predictive value of the ICD can help clinicians identify the high risk of LNM, so as to formulate individual treatment plan for patients.

Our study also has some limitations. First of all, the sample size of patients we included is small, which may affect the recognition efficiency. Although our research volume is relatively small, the incidence of LNM in OC is very high, considered that this is the major focus of this study, the sample size is sufficient. Secondly, this is a retrospective study, which may lead to bias in patient selection. Even so, we have proved that ICD is better than CA125 and D-dimer alone in predicting lymph node metastasis of ovarian cancer in both training and validation sets. ICD also has a certain reference value for predicting the prognosis of ovarian cancer. In the future, we will collect more data of ovarian cancer patients in medical centers to verify the significance of our model and expand the sample size for prospective cohort study to improve the limitations of this study.

In conclusion, the two medical centers selected in this study are very representative hospitals. Most ovarian cancer patients in this region and even in other regions will select these two hospitals for diagnosis and treatment. Interestingly, our study demonstrates that ICD is an indicator based on clinical serum tumor marker CA125 and D-dimer. Therefore, the ICD can be used as a convenient, reliable and economic biomarker to distinguish OC patients with or without LNM, which has reference significance for preoperative lymph node dissection strategy and early treatment intervention.

## Figures and Tables

**Table 1:** Characteristics of included patients (n=447)

Characteristics	Value (range or n%)
Number of patients	447 (100)
**Age**	
≤30 years	5 (1.11)
≥31, ≤40 years	16 (3.58)
≥41, ≤50 years	97 (21.70)
≥51, ≤60 years	150 (33.56)
≥61, ≤70 years	118 (26.40)
≥71, ≤80 years	54 (12.08)
≥81 years	7 (1.57)
**Menopausal status**	
Pre-menopausal	115 (25.73)
Post-menopausal	307 (68.68)
Hysterectomy	25 (5.59)
**FIGO stage**	
I-II	93 (20.81)
III-IV	354 (79.19)
**Histological type**	
Serous	163 (36.47)
Non-serous	284 (63.53)
**Degree of differentiation**	
Well + moderate	29 (6.49)
Poor	418 (93.51)
CA125, U/mL	491.3 (0.83-8067)
D-dimer, mg/L	1.69 (0.013-39.98)
**Lymph node metastasis**	
Yes	231 (51.68)
No	216 (48.32)
**Ascites**	
Yes	282 (63.09)
No	165 (36.91)

**Abbreviations:** CA125, cancer antigen 125; FIGO, International Federation of Gynecology and Obstetrics.

**Table 2:** Univariate and multivariate analyses for factors predicting LNM in OC

	Univariate		Multivariate	
	HR (95%CI)	P values	HR (95%CI)	P values
**Age**				
<58	Ref			
≥58	1.117 (0.770-1.619)	0.561		
**Menopausal status**				
Pre-menopausal	Ref			
Post-menopausal	0.681 (0.405-1.145)	0.148		
**CA125 (U/mL)**				
<490	Ref		Ref	
≥490	2.329 (1.593-3.403)	<0.001*	0.408 (0.170-0.977)	0.044*
**D-dimer (mg/L)**				
<1.69	Ref		Ref	
≥1.69	1.611 (1.109-2.341)	0.012*	1.897 (1.025-3.513)	0.042*
**ICD**				
≤0.5	Ref		Ref	
>0.5	2.814 (1.917-4.129)	<0.001*	2.651 (1.273-5.520)	0.009*
**HE4 (pmol/L)**				
<320	Ref			
≥320	4.056 (1.944-8.465)	<0.001*		
**Ascites**				
Yes	Ref			
No	1.133 (0.566-2.269)	0.725		
**Histological type**				
Serous	Ref			
Non-serous	1.494 (0.856-2.609)	0.159		
**Degree of differentiation**				
Well + moderate	Ref			
Poor	1.905 (0.521-6.962)	0.330		
**FIGO stage**				
I-II	Ref		Ref	
III-IV	2.901 (1.288-6.534)	0.009*	2.891 (1.291-6.473)	0.010*

**Abbreviations:** HE4, human epididymis secretory protein 4; CA125, cancer antigen 125; FIGO, International Federation of Gynecology and Obstetrics; ICD, indicator of CA125 combined with D-dimer.

## References

[1] Chen JP, Huang QD, Wan T, Tu H, Gu HF, Cao JY (2019). Combined score of pretreatment platelet count and CA125 level (PLT-CA125) stratified prognosis in patients with FIGO stage IV epithelial ovarian cancer. J Ovarian Res.

[2] Dochez V, Caillon H, Vaucel E, Dimet J, Winer N, Ducarme G (2019). Biomarkers and algorithms for diagnosis of ovarian cancer: CA125, HE4, RMI and ROMA, a review. J Ovarian Res.

[3] Charkhchi P, Cybulski C, Gronwald J, Wong FO, Narod SA, Akbari MR (2020). CA125 and Ovarian Cancer: A Comprehensive Review. Cancers (Basel).

[4] Huo Q, Xu C, Shao Y, Yu Q, Huang L, Liu Y (2021). Free CA125 promotes ovarian cancer cell migration and tumor metastasis by binding Mesothelin to reduce DKK1 expression and activate the SGK3/FOXO3 pathway. Int J Biol Sci.

[5] Hollis RL, Carmichael J, Meynert AM, Churchman M, Hallas-Potts A, Rye T (2019). Clinical and molecular characterization of ovarian carcinoma displaying isolated lymph node relapse. Am J Obstet Gynecol.

[6] Balbi G, Manganaro MA, Monteverde A, Landino I, Franzese C, Gioia F (2009). Ovarian cancer: lymph node metastases. Eur J Gynaecol Oncol.

[7] Seward SM, Winer I (2015). Primary debulking surgery and neoadjuvant chemotherapy in the treatment of advanced epithelial ovarian carcinoma. Cancer Metastasis Rev.

[8] Khiewvan B, Torigian DA, Emamzadehfard S, Paydary K, Salavati A, Houshmand S (2017). An update on the role of PET/CT and PET/MRI in ovarian cancer. Eur J Nucl Med Mol Imaging.

[9] Li C, Liu J, Lu R, Yu G, Wang X, Zhao Y (2011). AEG -1 overexpression: a novel indicator for peritoneal dissemination and lymph node metastasis in epithelial ovarian cancers. Int J Gynecol Cancer.

[10] Yang S, Li H, Liu Y, Ning X, Meng F, Xiao M (2013). Elevated expression of MAC30 predicts lymph node metastasis and unfavorable prognosis in patients with epithelial ovarian cancer. Med Oncol.

[11] Gu H, Tu H, Liu L, Liu T, Liu Z, Zhang W (2020). RSPO3 is a marker candidate for predicting tumor aggressiveness in ovarian cancer. Ann Transl Med.

[12] Yuan Q, Song J, Yang W, Wang H, Huo Q, Yang J (2017). The effect of CA125 on metastasis of ovarian cancer: old marker new function. Oncotarget.

[13] Theriault C, Pinard M, Comamala M, Migneault M, Beaudin J, Matte I (2011). MUC16 (CA125) regulates epithelial ovarian cancer cell growth, tumorigenesis and metastasis. Gynecol Oncol.

[14] Kim HS, Park NH, Chung HH, Kim JW, Song YS, Kang SB (2008). Significance of preoperative serum CA-125 levels in the prediction of lymph node metastasis in epithelial ovarian cancer. Acta Obstet Gynecol Scand.

[15] Sun J, Cui XW, Li YS, Wang SY, Yin Q, Wang XN (2020). The value of 18F-FDG PET/CT imaging combined with detection of CA125 and HE4 in the diagnosis of recurrence and metastasis of ovarian cancer. Eur Rev Med Pharmacol Sci.

[16] Chen Q, Zheng Y, Zhao H, Cai J, Wang L, Zhao J (2020). The combination of preoperative D-dimer and CA19-9 predicts lymph node metastasis and survival in intrahepatic cholangiocarcinoma patients after curative resection. Ann Transl Med.

[17] Gao XL, Wang SS, Cao DB, Liu W (2018). The role of plasma D-dimer levels for predicting lymph node and mediastinal lymph node involvement in non-small cell lung cancer. Clin Respir J.

[18] Chen F, Wang MJ, Li J, Yan CE, Han XH, Wu ZY (2015). Plasma D-dimer value as a predictor of malignant lymph node involvement in operable non-small cell lung cancer. Tumour Biol.

[19] Rong G, Zhang M, Xia W, Li D, Miao J, Wang H (2019). Plasma CADM1 promoter hypermethylation and D-dimer as novel metastasis predictors of cervical cancer. J Obstet Gynaecol Res.

[20] Ataseven B, Grimm C, Harter P, Prader S, Traut A, Heitz F (2014). Prognostic value of lymph node ratio in patients with advanced epithelial ovarian cancer. Gynecol Oncol.

[21] Nusbaum DJ, Mandelbaum RS, Machida H, Matsuzaki S, Roman LD, Sood AK (2020). Significance of lymph node ratio on survival of women with borderline ovarian tumors. Arch Gynecol Obstet.

[22] Cheng H, Peng J, Yang Z, Zhang G (2018). Prognostic significance of lymphadenectomyin malignant ovarian sex cord stromal tumor: A retrospective cohort study and meta-analysis. Gynecol Oncol.

[23] Komatsu H, Iida Y, Osaku D, Shimogai R, Chikumi J, Sato S (2021). Effects of pretreatment radiological and pathological lymph node statuses on prognosis in patients with ovarian cancer who underwent interval debulking surgery with lymphadenectomy following neoadjuvant chemotherapy. J Obstet Gynaecol Res.

[24] Eoh KJ, Yoon JW, Lee I, Lee JY, Kim S, Kim SW (2017). The efficacy of systematic lymph node dissection in advanced epithelial ovarian cancer during interval debulking surgery performed after neoadjuvant chemotherapy. J Surg Oncol.

[25] Rak J (2021). Cancer genes and blood clots. Blood.

[26] Kawai K, Watanabe T (2014). Colorectal cancer and hypercoagulability. Surg Today.

[27] Greco PS, Bazzi AA, McLean K, Reynolds RK, Spencer RJ, Johnston CM (2017). Incidence and Timing of Thromboembolic Events in Patients With Ovarian Cancer Undergoing Neoadjuvant Chemotherapy. Obstet Gynecol.

[28] Lima LG, Monteiro RQ (2013). Activation of blood coagulation in cancer: implications for tumour progression. Biosci Rep.

[29] Eriksson O, Thulin A, Asplund A, Hegde G, Navani S, Siegbahn A (2016). Cross-talk between the Tissue Factor/coagulation factor VIIa complex and the tyrosine kinase receptor EphA2 in cancer. BMC Cancer.

[30] Ghadhban BR (2018). Plasma d-dimer level correlated with advanced breast carcinoma in female patients. Ann Med Surg (Lond).

[31] Chen W, Zhong S, Shan B, Zhou S, Wu X, Yang H (2020). Serum D-dimer, albumin and systemic inflammatory response markers in ovarian clear cell carcinoma and their prognostic implications. J Ovarian Res.

[32] He X, Huang T, Xue Y, Zhang M, Liu Q, Wang Y (2019). Association of Preoperative Plasma D-dimmer and Fibrinogen and Renal Cell Carcinoma Outcome. J Cancer.

[33] Zhang C, Jia Y, Jia Y, Zhang X, Li K (2018). Prognostic and predictive value of plasma D-dimer levels in patients with small-cell lung cancer. Int J Clin Oncol.

[34] Chen Q, Zhao H, Wu J, Cai J, Li C, Zhao J (2019). Preoperative D-dimer and Gamma-Glutamyltranspeptidase Predict Major Complications and Survival in Colorectal Liver Metastases Patients After Resection. Transl Oncol.

[35] Blackwell K, Haroon Z, Broadwater G, Berry D, Harris L, Iglehart JD (2000). Plasma D-dimer levels in operable breast cancer patients correlate with clinical stage and axillary lymph node status. J Clin Oncol.

[36] Tomimaru Y, Yano M, Takachi K, Kishi K, Miyashiro I, Ohue M (2006). Plasma D-dimer levels show correlation with number of lymph node metastases in patients with esophageal cancer. J Am Coll Surg.

[37] Dai H, Zhou H, Sun Y, Xu Z, Wang S, Feng T (2018). D-dimer as a potential clinical marker for predicting metastasis and progression in cancer. Biomed Rep.

[38] Song X, Wang F, Shen H, Li J, Hu T, Yang Z (2019). [Correlation between Plasma D-dimer Count and Features ofNon-small Cell Lung Cancer]. Zhongguo Fei Ai Za Zhi.

[39] Rong G, Zhang M, Xia W, Li D, Miao J, Wang H (2019). Plasma CADM1 promoter hypermethylation and D-dimer as novel metastasis predictors of cervical cancer. J Obstet Gynaecol Res.

[40] Kitajima K, Ueno Y, Suzuki K, Kita M, Ebina Y, Yamada H (2012). Low-dose non-enhanced CT versus full-dose contrast-enhanced CT in integrated PET/CT scans for diagnosing ovarian cancer recurrence. Eur J Radiol.

